# Effect of Acetazolamide and Zoledronate on Simulated High Altitude-Induced Bone Loss

**DOI:** 10.3389/fendo.2022.831369

**Published:** 2022-02-09

**Authors:** Mikkel Bo Brent, Ulf Simonsen, Jesper Skovhus Thomsen, Annemarie Brüel

**Affiliations:** Department of Biomedicine, Aarhus University, Aarhus, Denmark

**Keywords:** diamox, high altitude, bone strength, bone loss, mountaineering

## Abstract

Exposure to hypobaric hypoxia at high altitude puts mountaineers at risk of acute mountain sickness. The carbonic anhydrase inhibitor acetazolamide is used to accelerate acclimatization, when it is not feasible to make a controlled and slow ascend. Studies in rodents have suggested that exposure to hypobaric hypoxia deteriorates bone integrity and reduces bone strength. The study investigated the effect of treatment with acetazolamide and the bisphosphonate, zoledronate, on the skeletal effects of exposure to hypobaric hypoxia. Eighty 16-week-old female RjOrl : SWISS mice were divided into five groups: 1. Baseline; 2. Normobaric; 3. Hypobaric hypoxia; 4. Hypobaric hypoxia + acetazolamide, and 5. Hypobaric hypoxia + zoledronate. Acetazolamide was administered in the drinking water (62 mg/kg/day) for four weeks, and zoledronate (100 μg/kg) was administered as a single subcutaneous injection at study start. Exposure to hypobaric hypoxia significantly increased lung wet weight and decreased femoral cortical thickness. Trabecular bone was spared from the detrimental effects of hypobaric hypoxia, although a trend towards reduced bone volume fraction was found at the L4 vertebral body. Treatment with acetazolamide did not have any negative skeletal effects, but could not mitigate the altitude-induced bone loss. Zoledronate was able to prevent the altitude-induced reduction in cortical thickness. In conclusion, simulated high altitude affected primarily cortical bone, whereas trabecular bone was spared. Only treatment with zoledronate prevented the altitude-induced cortical bone loss. The study provides preclinical support for future studies of zoledronate as a potential pharmacological countermeasure for altitude-related bone loss.

## 1 Introduction

Prolonged exposure to high altitude environments, i.e. above 2,500 to 3,000 meters, can result in acute mountain sickness (AMS). The risk of developing AMS is notably higher when mountaineers ascend rapidly without sufficient time for acclimatization to the diminished inspiratory oxygen pressure. The initial symptoms of AMS are headache, loss of appetite, fatigue, peripheral edema, sleep apnea, and general malaise (1). Although the initial symptoms of AMS are relatively mild, it may progress to life-threatening high altitude pulmonary edema (HAPE) (2) or high altitude cerebral edema (HACE) (3). Studies in rodents have suggested that exposure to high-altitude environments may affect the musculoskeletal system by reducing bone strength and inducing muscle atrophy (4, 5). However, observational follow-up studies in mountaineers are sparse, and only one study has measured bone mineral density (BMD) before and after expeditions to summit either Si Guang Feng (7,308 m) or Xixabangma (8,027 m). The study reported decreased BMD at the distal radius immediately after the expedition was completed, which was not fully recovered 12 months after returning to a habitual ambient pressure (6).

Several pharmaceutical countermeasures are routinely used to prevent or treat AMS (7). The Wilderness Medical Society recommends acetazolamide and dexamethasone to prevent AMS, although only acetazolamide facilitates acclimatization (8). Acetazolamide is a carbonic anhydrase inhibitor that causes bicarbonate diuresis, respiratory stimulation, and decreases cerebrospinal fluid production, and its effectiveness in the prevention of AMS has been established in multiple trials (9–12). Treatment of high altitude-related illnesses has previously mainly focused on pharmaceutical countermeasures against AMS, HAPE, and HACE, whereas treatments targeting the altitude-related deterioration in bone integrity is still uncharted territory.

Carbonic anhydrase is a family of enzymes that catalyze the interconversion between carbonic dioxide and water and the dissociated ions of carbonic acid (13) and is expressed in various cells, including osteoclasts (14). Osteoclasts use the cytosolic carbonic anhydrase II to produce protons that are transported through their ruffled border by H^+^-ATPases enabling intense acidification of the subjacent bone resorption pit (15). The acidification of the resorption pit is crucial to initiate inorganic mineral dissolution and successful bone resorption.

The effect of acetazolamide has previously been studied in bone cells (16, 17), as treatment for denervation-induced bone loss in rats (18), or to counteract post-menopausal osteoporosis in women (19), where it has been able to inhibit osteoclastic bone resorption and preserve bone mass. Still, to our knowledge, no study has investigated the skeletal effects of acetazolamide in rodents exposed to simulated high altitude or mountaineers participating in high altitude expeditions. Considering the crucial role of carbonic anhydrase to normal osteoclastic function and bone resorption, treatment with acetazolamide as a countermeasure against AMS might therefore have a pleasant side effect of mitigating altitude-induced bone loss. Knowledge of effective pharmacological countermeasures of bone loss is warranted since an increasing number of mountaineers are attempting to summit the highest mountains on earth each year, and many climbers are in their fifties or sixties (20, 21).

In the present study, we investigate whether treatment with acetazolamide alleviates the skeletal effects of exposure to simulated high altitude. In addition, the effect of acetazolamide is compared to that of the bisphosphonate zoledronate, which is currently the first-line therapy for most patients with osteoporosis (22).

## 2 Material and Methods

The study comprised eighty 16-week-old female mice (RjOrl : SWISS) purchased from Janvier Labs (Le Genest-Saint-Isle, France). At arrival the mice had a mean body weight of 34.8 ± 2.6 g. The animals were housed groupwise (*n* = 6/cage) at the animal facility at Aarhus University, Denmark at a constant temperature of 20°C and computer-controlled light/dark cycle (12/12 h). All cages were standard plastic cages (1290D, Eurostandard Type III, Tecniplast, Milan, Italy) with a floor area of 820 cm^2^ (425 mm × 266 mm × 155 mm), equipped with rodent nesting material made of kraft paper (Sizzle Nest, Datesand, UK), play tunnels, and wood gnawing blocks. During the study, all mice had unrestricted access to standard pelleted rodent chow and tap water (1324 maintenance diet for rats and mice, Altromin, Lage, Germany). Seven days before study start, the mice were stratified according to their body weight into five groups (*n* = 16/group): 1. Baseline; 2. Normobaric (Normo); 3. Hypobaric hypoxia (Hypo); 4. Hypobaric hypoxia + acetazolamide (Hypo + AZ), and 5. Hypobaric hypoxia + zoledronate (Hypo + ZOL).

Acetazolamide (A6011, Sigma-Aldrich, St. Louis, MO, USA) was mixed with the drinking water, and 2% sucrose was added (145 mg acetazolamide/liter) before the solution was filled into custom-made light-shielded black drinking bottles. Chow consumption and water intake was monitored weekly, and the concentration of acetazolamide in the drinking water was adjusted to ensure a target dose of approximately 50 mg/kg/day. Acetazolamide was administered until the end of the study. Only animals treated with acetazolamide received sucrose in their drinking water to encourage consumption. Zoledronate (100 μg/kg, Fresenius Kabi, Bad Homburg vor der Höhe, Germany) was injected subcutaneously (s.c.) once at the study start.

**Figure 1:**
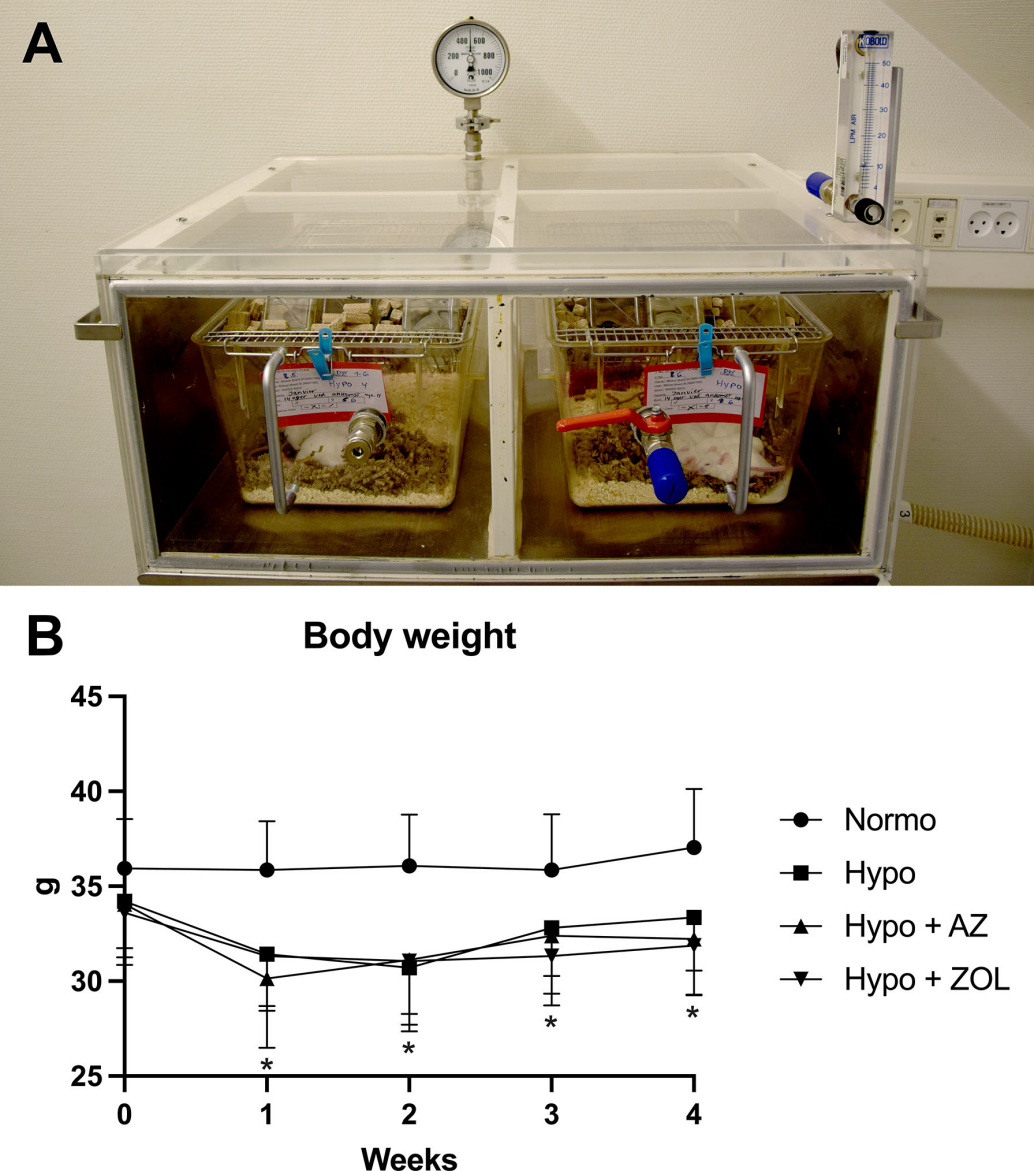
**(A)** Setup of the hypobaric chambers used to expose mice to simulated high altitude (500 mbar, which approximately corresponds to the ambient pressure at 5,500 m above sea level). The pressure was controlled using an electric vacuum-pump located in another room to eliminate noise. **(B)** Development of body weight throughout the study. All data were analyzed using a one-way ANOVA. Data are presented as mean ± SD and *n* = 15–16/group. *p < 0.05 vs. Normo.

Mice allocated to hypobaric hypoxia were housed at 500 mbar (corresponding to an altitude of 5,500 m or approximately the barometric pressure at Mount Everest Base Camp), whereas mice in the Normobaric group were housed at sea level atmospheric pressure (**Figure 1A**). The hypobaric environment was only interrupted for one hour once weekly for cleaning and replacing water and chow. Mice allocated to hypobaric hypoxia were acclimatized for three days before study start, to enable a gradual adaptation to the lower ambient pressure. The animal model of hypobaric hypoxia has previously been used and described in detail (5).

In order to assess bone formation throughout the study, all animals except the Baseline group, were injected s.c. with tetracycline (20 mg/kg, T3383, Sigma-Aldrich, St. Louis, MO, USA) and alizarin (20 mg/kg, A3882, Sigma-Aldrich, St. Louis, MO, USA) one and two weeks before sacrifice, respectively.

Mice allocated to the Baseline group were sacrificed at study start to establish the skeletal status at baseline, while the remaining mice were sacrificed after four weeks (5). All mice were sacrificed under general anesthesia by inhalation of isoflurane (Attane Vet, ScanVet, Fredensborg, Denmark), and immediately thereafter, tissue was extracted. One mouse allocated to the Normobaric group and one to the Hypobaric group died unexpectedly before the study finished.

All animal procedures were approved by the Danish Animal Experiment Inspectorate (2018–15–0201–01436) and reported in accordance with the ARRIVE guidelines (23). Animal welfare was assessed daily by veterinarians, animal technicians, or the investigators.

Animal handling, injection of fluorochromes, treatment administration, dual-energy X-ray absorptiometry, micro computed tomography, and mechanical testing were not performed blinded to group allocations. However, both dynamic and static bone histomorphometry were performed blinded to group allocation. In order to reduce the impact of experimental bias, all laboratory procedures were conducted across group allocations, instead of groupwise.

### 2.1 Tissue Extraction

Blood was drawn from the vena cava, and immediately after sacrifice, the lungs and heart were removed and weighed using a digital scale (Mettler AT250, Columbus, OH, USA). Right and left atria of the heart were carefully removed before the free wall of the right ventricle (RV) was separated from the left ventricle and septum (LV+S). Each part of the heart was weighed, and the right ventricle to the left ventricle plus septum weight ratio (RV/(LV+S)) was determined and used to assess right ventricular hypertrophy as previously described (24). The wet weight of the lungs was used as a surrogate marker for pulmonary edema (25, 26).

The right rectus femoris muscle was removed, weighed, and immersion-fixed in 0.1 M sodium phosphate-buffered formaldehyde (4% formaldehyde, pH 7.0) (27).

Both femora, tibia, and L4 vertebra were isolated and any remaining soft tissue were carefully removed. The right femur and tibia and L4 were stored in Ringer’s solution at −20°C, while the left femur was immersion-fixed in 0.1 M sodium phosphate-buffered formaldehyde (4% formaldehyde, pH 7.0) for 48 h and then stored in 70% ethanol (5). Bone lengths of the right femur and tibia were determined using a digital sliding caliper.

### 2.2 Whole Muscle and Muscle Cell Cross-Sectional Area

The rectus femoris muscles were halved at the midpoint, placed on a flat-bed image scanner (Perfection 3200 Photo; Seiko Epson, Nagano, Japan), and scanned at 300 DPI to determine the whole muscle cross-sectional area (CSA) (28). CSA was estimated in Adobe Photoshop 2021 (San Jose, California, USA) by contouring the muscle CSA.

The halved rectus femoris muscle was then immersion-fixed in 0.1 M sodium phosphate- buffered formaldehyde (4% formaldehyde, pH 7.0) and embedded in plastic-based 2-hydroxyethyl methacrylate (Technovit 7100, Heraeus Kulzer, Wehrheim, Germany). On a microtome (Jung RM2065; Leica Instruments, Nussloch, Germany), the embedded muscles were cut into 2-μm-thick sections and stained with Masson’s trichrome. The sections were used to determine myofiber CSA using a light microscope (Nikon Eclipse i80, Tokyo, Japan) at a final magnification of ×1190 (29). On average, 234 myofiber profiles were counted per sample to determine the average myofiber CSA.

### 2.3 Hematocrit

Blood was collected in microcapillary tubes and centrifuged (iFuge HCT, Neuation, Gujarat, India) at 14,000 relative centrifugal force (RCF) for 5 min. Then, the hematocrit was estimated using a manual microhematocrit reader as previously described (5).

### 2.4 Dual-Energy X-ray Absorptiometry

In order to determine areal bone mineral density (aBMD) and bone mineral content (BMC), the right femur and tibia were scanned by dual-energy X-ray absorptiometry (DXA) (pDEXA Sabre XL; Norland Stratec, Pforzheim, Germany) at a pixel size of 0.1 mm × 0.1 mm and a scan speed of 3.0 mm/s (5).

### 2.5 Micro Computed Tomography

**Figure 2:**
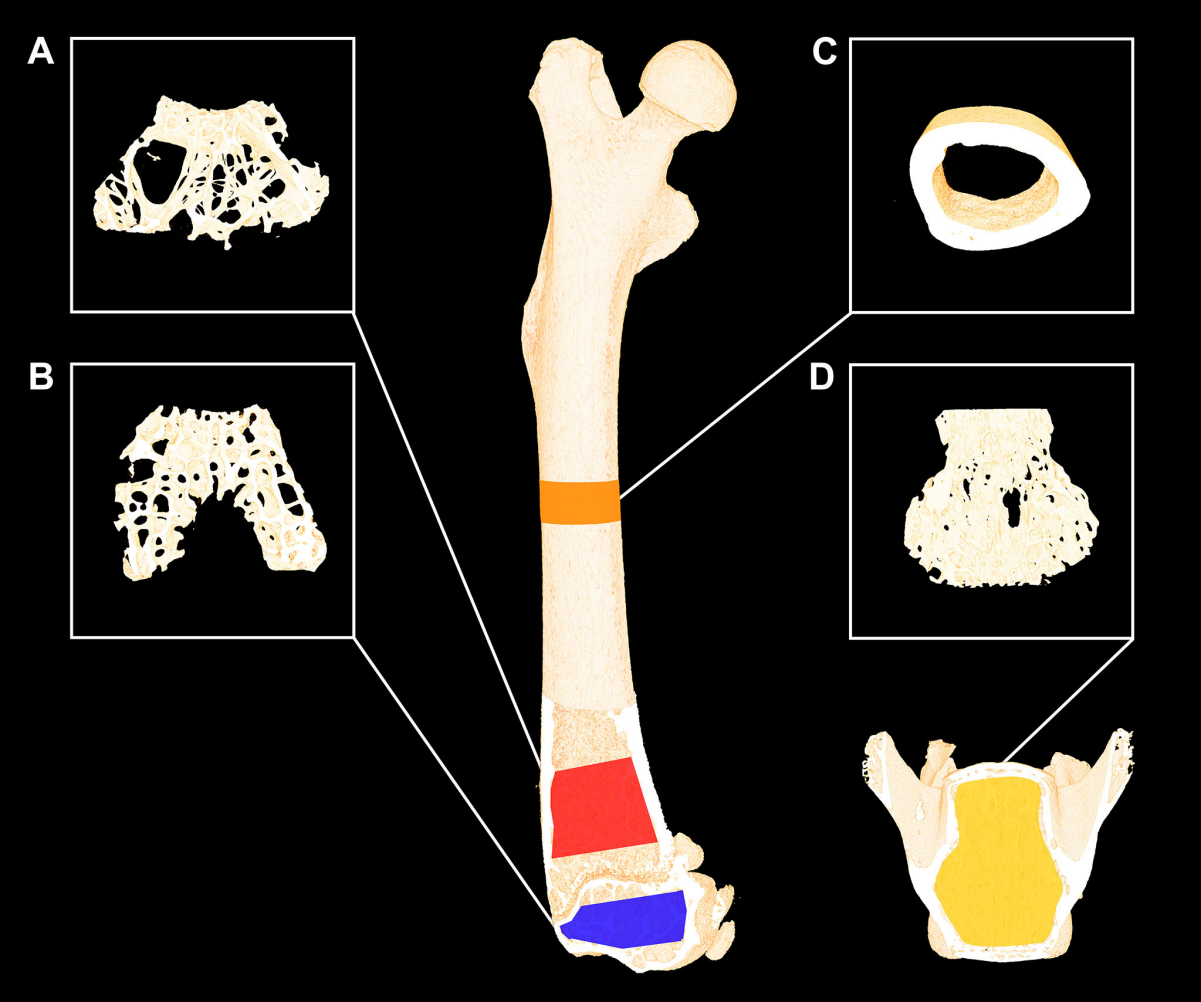
Femoral and L4 bone sites investigated with µCT using an isotropic voxel size of 3.5 µm. **(A)** Red: 1,000-μm-high volume of interest (VOI) at the distal femoral metaphysis. **(B)** Blue: 490-μm-high VOI at the distal femoral epiphysis. **(C)** Orange: 820-μm-high VOI at the femoral mid-diaphysis. **(D)** Yellow: 2000-µm-high VOI at the L4 vertebral body. Dimensions are not to scale.

Trabecular microstructure and cortical morphology were analyzed using a desktop micro computed tomography (μCT) scanner (Scanco μCT 35, Scanco Medical AG, Brüttiselen, Switzerland) (**Figure 2**). The distal femoral metaphysis, femoral epiphysis, femoral mid-diaphysis, and L4 were scanned in high-resolution mode (1000 projections/180°), at an isotopic voxel size of 3.5 μm, an X-ray tube voltage of 55 kVp, a current of 145 μA, and an integration time of 800 ms. Beam hardening effects were reduced using a 0.5 mm aluminum filter (5, 30).

The distal femoral metaphysis was analyzed using a 1000-μm-high volume of interest (VOI), the femoral epiphyseal VOI was approximately 330-μm-high, and L4 was analyzed using an approximately 3,000-μm-high VOI. All these VOIs contained trabecular bone only as previously described in detail (5, 30). The cortical bone of the femoral mid-diaphysis was analyzed using an 820-μm-high VOI centered on the mid-point of the femur (5, 30).

A Gaussian filter (σ = 0.8 and support = 1) were used to low-pass filter the 3D data and segmentation was conducted with a global fixed threshold filter of 548 mg HA/cm^3^.

### 2.6 Mechanical Testing

Bone strengths of the femoral mid-diaphysis, femoral neck, and vertebral body of L4 were determined using a material testing machine (Instron model 5566, United Kingdom) as previously described (29, 31, 32). In brief, the femoral bone was placed with the anterior side facing up on two rounded supporting bars separated by 7.14 mm for the 3-point bending test. Vertical load was applied to the femoral mid-diaphysis with a third rounded bar at a constant deformation rate of 2 mm/min until fracture. Then, the proximal part of the femur was placed in a custom-made fixation device exposing the femoral neck and vertical load was applied to the femoral head neck using a steel cylinder at a constant deformation rate of 2 mm/min until fracture. After the vertebral disc and processi were removed, the corpus of L4 was compression tested in a similar way. The highest force achieved during the mechanical testing determined from the load-displacement data was considered maximum bone strength. Mechanical testing data were analyzed using in-house developed software.

### 2.7 Cortical Bone Specimens and Microscopy

Using a precision saw (EXAKT Apparatebau, Norderstedt, Germany), a 200-μm-thick cross-sectional slice of the right femoral mid-diaphysis was prepared and mounted with Pertex on a glass slide for dynamic bone histomorphometry as previously described (27). Embedded undecalcified in methyl methacrylate (MMA), the distal part of the left femur was cut in 7-μm-thick longitudinal sections on a microtome (Jung RM2065; Leica Instruments, Nussloch, Germany). The sections were either left unstained for dynamic bone histomorphometry, stained with Masson Goldner trichrome to asses osteoblast and osteoid-covered surfaces, or stained for tartrate-resistant acid phosphatase (TRAP) and counterstained with aniline blue to detect osteoclasts (33). A microscope (Nikon Eclipse i80, Tokyo, Japan) able to project live images to a computer equipped with the Visiopharm stereology software was used for the histological assessment. All histological assessments using optical microscopy were conducted at a final magnification of ×1190 and the fields of view for longitudinal sections were sampled covering 100% of the region of interest (ROI).

### 2.8 Dynamic Bone Histomorphometry

The unstained sections of the left distal femoral metaphysis and right femoral mid-diaphysis were used for dynamic bone histomorphometry to determine mineralizing surfaces (MS/BS), mineral apposition rate (MAR), and bone formation rate (BFR/BS) as previously described (5, 30). An imputed value of zero was used for MAR in case of no double labels (34). MS/BS, MAR, and BFR/BS were quantified and calculated in accordance with the current ASBMR Histomorphometry Nomenclature Committee guideline (34).

For the femoral mid-diaphysis, labels at the periosteal and endocortical bone surface were counted using a 24-arm radiating grid. For the distal femoral metaphysis, a 1000-μm-high ROI was delineated along the endocortical edge starting 300 μm above the growth plate, containing trabecular bone only (5, 30).

### 2.9 Bone Cells

The Masson-Goldner trichrome-stained sections of the left femoral metaphysis were used to estimate osteoblast-covered surfaces (Ob.S/BS) and osteoid-covered surfaces (OS/BS). Osteoblasts were defined as cuboidal cells with a single nucleus residing on an intact bone surface. Osteoid were defined as the red unmineralized matrix at the very edge of the bone surface. The sections of the left femoral metaphysis stained for TRAP were used to estimate osteoclast-covered surfaces (Oc.S/BS). Osteoclasts were defined as multinucleated TRAP-positive cells residing on an intact bone surface (34). Osteoblasts, osteoid, and osteoclasts were estimated using a 1000-μm-high ROI starting 300 μm above the growth plate, containing trabecular bone only (5).

### 2.10 Statistics

Data were analyzed using GraphPad Prism 9.1.1. (GraphPad Software, San Diego, CA, USA). Normality of data were assessed by visual inspection of Q–Q plots and the D’Agostino-Pearson normality test. A one-way analysis of variance (ANOVA) followed by a *post-hoc* Holm-Sidak test was used, whenever normal distribution requirements were met. If the data were not normally distributed, a Kruskal–Wallis one-way ANOVA on ranks was performed, followed by a *post-hoc* Dunn’s test. Results were defined as statistically significant if the *p*-values were below 0.05. All animals were investigated with all of the methods described above. All animals were included in the statistical analyses, except Baseline.

An *a priori* sample size calculation (power = 0.8) on female RjOrl : SWISS mice from a previous study using hypobaric chambers showed that an 8% difference in femoral aBMD can be demonstrated between groups with n = 16 animals (5).

## 3 Results

### 3.1 Body Weight, Chow Consumption, and Cardiopulmonary Effects

Hypobaric hypoxia significantly decreased daily chow consumption (–18%, *p* < 0.001) resulting in a substantial decrease in body weight (–10%, *p* = 0.003) after four weeks compared with mice housed at normobaric ambient pressure (**Figure 1B** and **Table 1**). The body weight of mice exposed to hypobaric hypoxia was significantly lower than that of normobaric mice throughout the study. After an initial loss of body weight during week one, hypobaric hypoxia mice gained weight in parallel to the normobaric mice.

**Table 1:** Number of animals per group, mean daily chow consumption per mouse, right ventricle to left ventricle and septum [RV/(LV+S)] weight ratio, lungs wet weight, hematocrit, muscle mass, muscle cross-sectional area (CSA), and myofiber CSA of mice housed at normobaric ambient pressure (Normo) or hypobaric pressure (Hypo) at 500 mbar for four weeks and treated with acetazolamide (AZ) or zoledronate (ZOL).

	Baseline	Normo	Hypo	Hypo + AZ	Hypo + ZOL
Number of animals (*n*)	16	15	15	16	16
Chow consumption (g/day)	−	4.78 ± 0.17	3.93^*^ ± 0.72	3.11^*,#^ ± 0.68	3.33^*,#^ ± 0.52
RV/(LV+S) (%)	25.1 ± 5.37	22.3 ± 7.72	36.5^*^ ± 9.80	36.2^*^ ± 6.72	41.1^*^ ± 5.58
Lungs (mg)	235 ± 24.4	253 ± 22.9	313^*^ ± 20.8	304^*^ ± 34.5	313^*^ ± 28.6
Hematocrit (%)	43.6 ± 2.28	40.9 ± 2.19	59.5^*^ ± 5.76	64.2^*,#^ ± 4.92	64.3^*,#^ ± 6.58
Rectus femoris muscle mass (mg)	88.4 ± 7.18	93.7 ± 8.30	89.9 ± 10.1	85.9 ± 10.1	84.8 ± 9.79
Rectus femoris muscle CSA (mm^2^)	13.6 ± 1.64	14.5 ± 2.27	12.7^*^ ± 1.40	12.2^*^ ± 2.01	12.2^*^ ± 1.42
Rectus femoris myofiber CSA (μm^2^)	2706 ± 332	2804 ± 386	2427^*^ ± 155	2425^*^ ± 295	2439^*^ ± 307

One mouse allocated to the Normobaric group and one to the Hypobaric group died before the study finished. All data were analyzed using a one-way ANOVA, except for rectus femoris myofiber CSA where the non-parametric Kruskal–Wallis test was used. Data are presented as mean ± SD and n = 15–16/group. *p < 0.05 vs. Normo and ^#^p < 0.05 vs. Hypo.

Right ventricular size (+70%, *p* < 0.001), wet weights of the lungs (+23%, *p* < 0.001), and hematocrit (+53%, *p* < 0.001) were profoundly increased for all mice exposed to hypobaric hypoxia compared with normobaric mice (**Table 1**).

These findings are consistent with the expected physiological response to a high-altitude environment with reduced oxygen availability.

### 3.2 Rectus Femoris Muscle

No significant difference between any of the groups was found for rectus femoris muscle mass. However, a non-significant trend towards decreased muscle mass was found for all mice assigned to hypobaric hypoxia (**Table 1**).

Rectus femoris whole muscle CSA (–12%, *p* = 0.034) and myofiber CSA (–13%, *p* = 0.029) were significantly reduced in mice exposed to hypobaric hypoxia compared with mice at normobaric ambient pressure. Neither acetazolamide, nor zoledronate affected whole muscle CSA or myofiber CSA compared with hypobaric hypoxia (**Table 1**).

These findings suggest detrimental effects of hypobaric hypoxia on muscle tissue and affects not only the CSA of the whole muscle but also the CSA of the individual myofibers.

### 3.3 DXA

Hypobaric hypoxia did not reduce bone mineral density, bone mineral content, or bone length of either femur or tibia compared with normobaric mice (**Table 2**).

**Table 2:** Whole femoral and tibial areal bone mineral density (aBMD), bone mineral content (BMC), and bone length of mice housed at normobaric ambient pressure (Normo) or hypobaric pressure (Hypo) at 500 mbar for four weeks and treated with acetazolamide (AZ) or zoledronate (ZOL). All data were analyzed using a one-way ANOVA, except for femoral BMC and tibial length where the non-parametric Kruskal–Wallis test were used.

	Baseline	Normo	Hypo	Hypo + AZ	Hypo + ZOL
**Femur**					
aBMD (mg/cm^2^)	88.8 ± 4.92	84.1 ± 6.42	83.1 ± 4.78	80.7 ± 9.47	88.4^†^ ± 7.49
BMC (mg)	37.5 ± 2.78	36.0 ± 3.67	36.3 ± 2.67	34.4 ± 4.87	37.4 ± 3.90
Bone length (mm)	16.2 ± 0.44	16.5 ± 0.45	16.6 ± 0.53	16.5 ± 0.59	16.5 ± 0.66
**Tibia**					
aBMD (mg/cm^2^)	73.0 ± 4.09	71.4 ± 5.19	70.9 ± 5.56	67.6 ± 6.04	74.4^†^ ± 5.65
BMC (mg)	27.1 ± 2.60	27.0 ± 2.83	26.9 ± 2.76	25.0 ± 2.82	27.5 ± 2.91
Bone length (mm)	19.4 ± 0.50	19.6 ± 0.40	19.4 ± 0.91	19.4 ± 0.85	19.5 ± 0.50

Data are presented as mean ± SD and n = 15–16/group. ^†^p < 0.05 vs. Hypo + AZ.

Zoledronate significantly increased femoral and tibial bone mineral density (+6%, *p* = 0.026 and +5%, *p* = 0.007), but not bone mineral content, compared to non-treated hypobaric hypoxia mice, respectively. Treatment with acetazolamide did not affect either bone mineral density or bone mineral content compared with mice exposed to hypobaric hypoxia alone (**Table 2**).

These findings suggest bone mineral density, bone mineral content, and bone length are spared from the detrimental effects of hypobaric hypoxia. In addition, zoledronate was able to increase bone mineral density despite the hypobaric environment, whereas acetazolamide was not.

### 3.4 μCT

#### 3.4.1 Trabecular Microstructure

At the distal femoral metaphysis, no harmful effects of hypobaric hypoxia were found for bone volume fraction, trabecular number, trabecular spacing, or any other microstructural parameter assessed (**Table 3**). In contrast, hypobaric hypoxia significantly increased trabecular thickness (+13%, *p* = 0.047) compared with normobaric mice. Acetazolamide significantly decreased trabecular thickness (–12%, *p* = 0.028) compared with non-treated hypobaric hypoxia mice, as the only observable effect.

**Table 3:** Microstructural properties of the distal femoral metaphysis and epiphysis and L4 of mice housed at normobaric ambient pressure (Normo) or hypobaric pressure (Hypo) at 500 mbar for four weeks and treated with acetazolamide (AZ) or zoledronate (ZOL).

	Baseline	Normo	Hypo	Hypo + AZ	Hypo + ZOL
**Femoral metaphysis**					
BV/TV (%)	13.5 ± 4.78	8.87 ± 3.63	8.50 ± 4.36	9.33 ± 3.33	13.9^*,#,†^ ± 4.22
Tb.Th (µm)	54.7 ± 4.97	50.0 ± 4.61	56.5^*^ ± 7.33	49.5^#^ ± 8.27	55.1 ± 5.68
Tb.N (mm^−1^)	3.38 ± 0.86	2.53 ± 0.59	2.34 ± 0.79	2.74 ± 0.73	3.18^#^ ± 0.78
Tb.Sp (µm)	341 ± 96.1	439 ± 97.8	512 ± 171	416 ± 125	362^#^ ± 116
CD (mm^−3^)	185 ± 84.4	130 ± 64.5	105 ± 68.0	145 ± 90.9	169 ± 75.3
SMI	0.89 ± 0.50	1.06 ± 0.46	1.09 ± 0.36	1.13 ± 0.26	0.80 ± 0.37
vBMD (mg/cm^3^)	161 ± 57.7	106 ± 46.3	101 ± 53.5	113 ± 38.5	173^*,#,†^ ± 50.7
TMD (mg/cm^3^)	991 ± 11.5	978 ± 16.6	977 ± 24.5	973 ± 24.4	1021^*,#,†^ ± 14.2
**Femoral epiphysis**					
BV/TV (%)	34.5 ± 5.55	30.0 ± 4.69	25.4 ± 5.38	27.9 ± 5.09	33.2^#^ ± 7.52
Tb.Th (µm)	65.4 ± 4.53	64.3 ± 4.93	66.9 ± 5.82	61.0 ± 8.69	67.2^†^ ± 4.65
Tb.N (mm^−1^)	7.86 ± 0.77	7.03 ± 0.62	6.71 ± 0.48	6.92 ± 0.86	7.44 ± 0.97
Tb.Sp (µm)	161 ± 18.3	177 ± 18.0	188 ± 16.1	177 ± 21.0	165^#^ ± 19.0
CD (mm^−3^)	301 ± 71.8	216 ± 40.4	187 ± 60.6	245 ± 67.3	243 ± 91.6
SMI	−0.59 ± 0.37	−0.16 ± 0.30	−0.01 ± 0.39	−0.15 ± 0.29	−0.46^#^ ± 0.43
vBMD (mg/cm^3^)	418 ± 63.8	366 ± 54.4	311 ± 65.8	341 ± 59.4	410^#,†^ ± 88.1
TMD (mg/cm^3^)	1062 ± 9.68	1056 ± 10.6	1052 ± 16.6	1048 ± 18.5	1079^*,#,†^ ± 10.7
**L4**					
CD (mm^−3^)	360 ± 88.8	286 ± 71.2	233 ± 80.2	273 ± 80.7	282 ± 122
SMI	−0.27 ± 0.40	0.02 ± 0.36	0.30 ± 0.39	0.14 ± 0.23	−0.16^#^ ± 0.49
vBMD (mg/cm^3^)	303 ± 49.8	252 ± 42.3	207 ± 55.3	236 ± 48.2	293^#,†^ ± 75.1
TMD (mg/cm^3^)	968 ± 16.5	952 ± 12.3	944 ± 21.6	951 ± 16.5	981^*,#,†^ ± 18.4

Bone volume fraction (BV/TV), trabecular thickness (Tb.Th), trabecular spacing (Tb.Sp), trabecular number (Tb.N), connectivity density (CD), structure model index (SMI), volumetric bone mineral density (vBMD), and tissue mineral density (TMD). All data were analyzed using a one-way ANOVA, except for femoral metaphyseal Tb.Sp, CD, vBMD, and TMD, and femoral epiphyseal Tb.Sp, CD, SMI, and TMD, and L4 CD where the non-parametric Kruskal–Wallis test were used. Data are presented as mean ± SD and n = 15–16/group. *p < 0.05 vs. Normo, ^#^p < 0.05 vs. Hypo, and ^†^p < 0.05 vs. Hypo + AZ.

At the distal femoral metaphysis, treatment with zoledronate significantly increased bone volume fraction (+64%, *p* = 0.02), trabecular number (+36%, *p* = 0.013), volumetric bone mineral density (+71%, *p* = 0.005), and tissue mineral density (5%, *p* < 0.001) and significantly decreased trabecular spacing (–29%, *p* = 0.027) compared with non-untreated hypobaric hypoxia mice. In addition, the zoledronate induced increase in bone volume fraction (+57%, *p* = 0.003 and +49%, *p* = 0.006), volumetric bone mineral density (+63%, *p* = 0.005 and +53%, *p* = 0.019), and tissue mineral density (+4%, *p* < 0.001 and +5%, *p* < 0.001) was significantly above that of both non-treated normobaric mice and acetazolamide-treated mice, respectively.

At the distal femoral epiphysis, no significant detrimental effects of hypobaric hypoxia were found for any trabecular microstructural parameter investigated compared with normobaric mice (**Table 3**). However, a non-significant trend was found towards decreased bone volume fraction (–15%, *p* = 0.128) in mice exposed to hypobaric hypoxia compared with normobaric mice. Treatment with acetazolamide did not evoke any differences in trabecular microstructure compared with both normobaric and hypobaric mice. Zoledronate significantly increased bone volume fraction (+31%, *p* = 0.003), volumetric bone mineral density (+32%, *p* = 0.001), and tissue mineral density (+3%, *p* < 0.001), and reduced trabecular spacing (–12%, *p* = 0.026) and structure model index compared with non-treated hypobaric mice. In addition, the zoledronate-induced increase in volumetric bone mineral density (+17%, *p* = 0.030) and tissue mineral density (+3%, *p* < 0.001) was significantly higher than in mice treated with acetazolamide.

**Figure 3:**
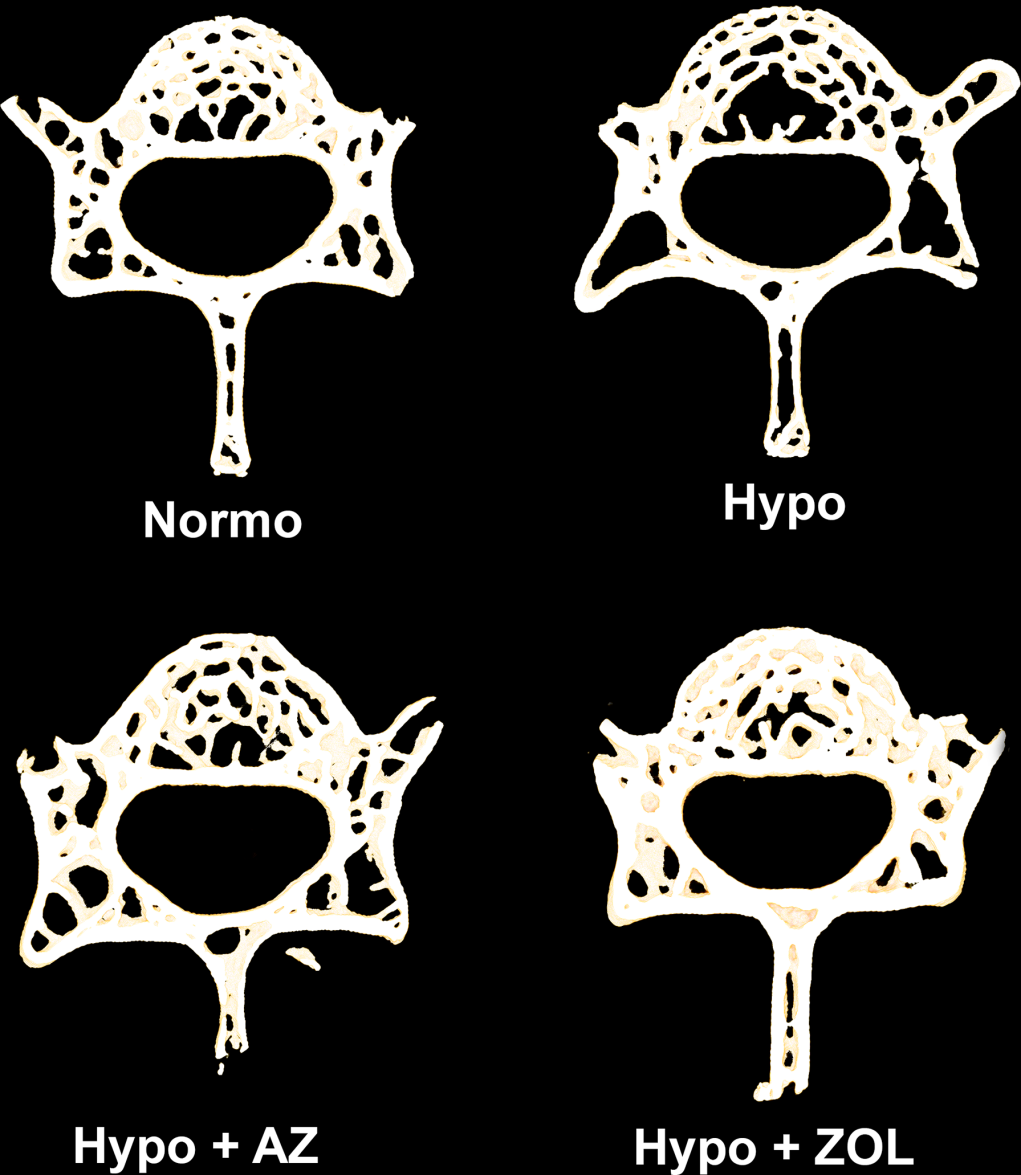
105-μm-thick representative horizontal sections of the L4 vertebral body from mice housed at normobaric pressure (Normo) or exposed to hypobaric pressure (Hypo) at 500 mbar for four weeks and treated with acetazolamide (AZ) or zoledronate (ZOL). Treatment with ZOL significantly increased vertebral bone volume fraction (BV/TV) compared with Hypo.

**Figure 4:**
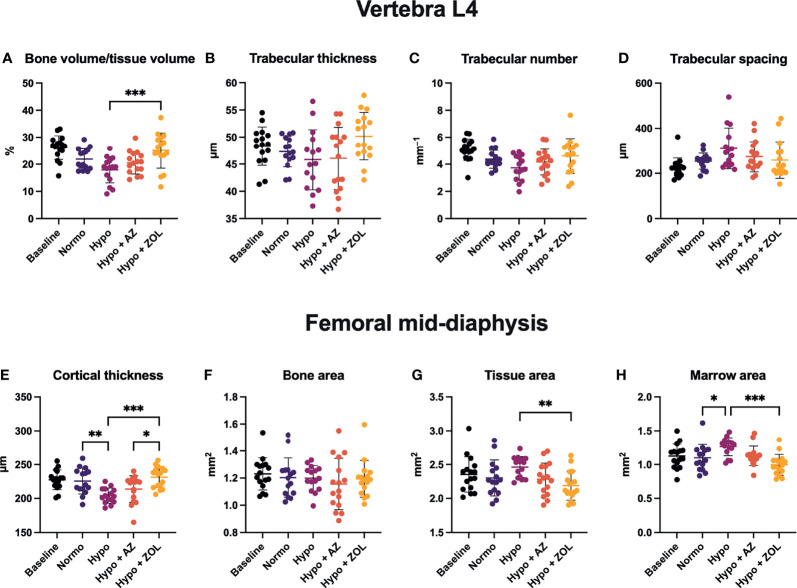
Trabecular microstructure **(A–D)** and cortical morphology **(E–H)** of mice housed at normobaric ambient pressure (Normo) or hypobaric pressure (Hypo) at 500 mbar for four weeks and treated with acetazolamide (AZ) or zoledronate (ZOL). All data were analyzed using a one-way ANOVA, except for L4 trabecular spacing and femoral mid-diaphyseal bone area and marrow area where the non-parametric Kruskal–Wallis test were used. Data are presented as mean ± SD and n = 15–16/group. *p < 0.05, **p < 0.01, and ***p < 0.001.

At L4, hypobaric hypoxia did not have any significantly negative effects on the trabecular microstructure (**Figures 3**, **4** and **Table 3**). However, a non-significant trend towards a decreased bone volume fraction (–18%, *p* = 0.124) was found in mice subjected to hypobaric hypoxia compared with normobaric mice. No effect of acetazolamide was found on any trabecular microstructural parameter investigated compared with both normobaric and hypobaric mice. In contrast, treatment with zoledronate significantly increased bone volume fraction (+40%, *p* = 0.001), volumetric bone mineral density (+32%, *p* = 0.001), and tissue mineral density (+3%, *p* < 0.001) compared with non-treated mice exposed to hypobaric hypoxia.

Overall, these findings suggest few negative effects of hypobaric hypoxia on the vertebral trabecular microstructure. Acetazolamide had no positive effects, while the anti-resorptive effect of zoledronate was apparent despite concomitant exposure to hypobaric hypoxia.

#### 3.4.2 Cortical Morphology

At the femoral mid-diaphysis, exposure to hypobaric hypoxia significantly decreased cortical thickness (–10%, *p* = 0.040) and increased marrow area (+15%, *p* = 0.043) compared with normobaric mice (**Figures 4E, H** **)**. Treatment with acetazolamide did not affect any cortical bone parameter assessed compared with both normobaric and hypobaric mice. Zoledronate significantly increased cortical thickness (+13%, *p* < 0.001) and reduced both tissue area (–11%, *p* = 0.007) and marrow area (–21%, *p* < 0.001) compared with non-treated mice exposed to hypobaric hypoxia. The cortical thickness of zoledronate-treated mice (+8%, *p* = 0.017) was also significantly above that of mice treated with acetazolamide.

These findings suggest that the detrimental effects of hypobaric hypoxia on cortical thickness develop due to an increased endocortical bone resorption.

### 3.5 Bone Strength

There was no significant difference in bone strength at the femoral mid-diaphysis or femoral neck between any of the groups (**Table 4**).

**Table 4:** Maximum bone strength at femoral mid-diaphysis, femoral neck, and L4 of mice housed at normobaric ambient pressure (Normo) or hypobaric pressure (Hypo) at 500 mbar for four weeks and treated with acetazolamide (AZ) or zoledronate (ZOL).

	Baseline	Normo	Hypo	Hypo + AZ	Hypo + ZOL
Femoral mid-diaphysis (N)	27.5 ± 3.22	25.9 ± 4.17	26.9 ± 2.89	25.5 ± 4.73	26.7 ± 4.03
Femoral neck (N)	23.6 ± 3.26	23.3 ± 3.19	21.9 ± 2.52	20.2 ± 4.18	22.3 ± 3.20
L4 (N)	29.8 ± 6.89	29.2 ± 6.80	25.6 ± 10.1	27.9 ± 9.24	34.1 ± 13.8

All data were analyzed using a one-way ANOVA, except for femoral neck strength where the non-parametric Kruskal–Wallis test was used. Data are presented as mean ± SD and n = 15–16/group.

### 3.6 Dynamic Bone Histomorphometry and Bone Cells

#### 3.6.1 Trabecular Bone

**Figure 5:**
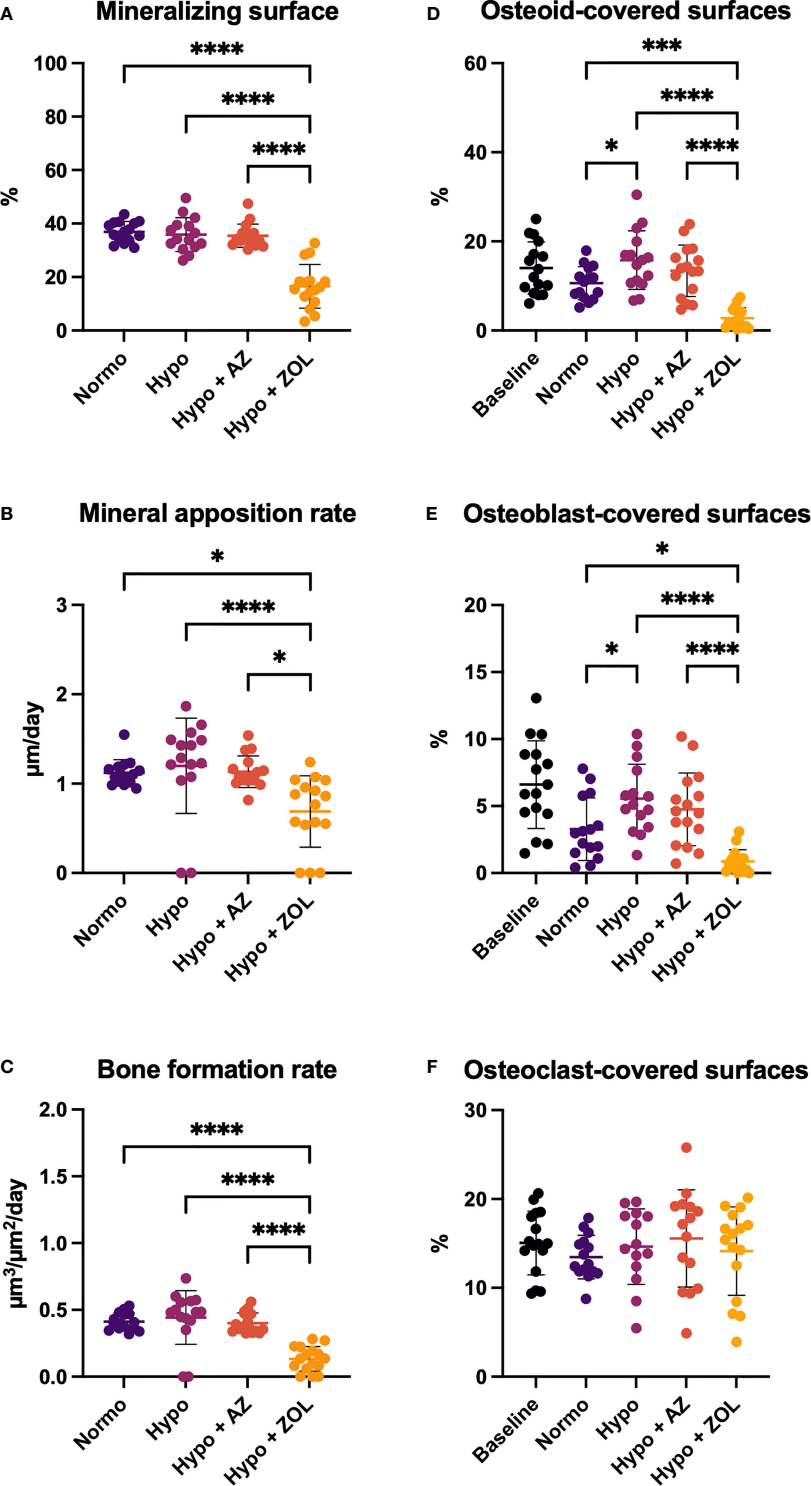
Distal femoral trabecular bone parameters determined by dynamic bone histomorphometry **(A–C)** and osteoid **(D)** and bone cells quantification **(E, F)** of mice housed at normobaric ambient pressure (Normo) or hypobaric pressure (Hypo) at 500 mbar for four weeks and treated with acetazolamide (AZ) or zoledronate (ZOL). All data were analyzed using a one-way ANOVA, except for mineralizing surface and mineral apposition rate where the non-parametric Kruskal–Wallis test were used. Data are presented as mean ± SD and n = 15–16/group. *p < 0.05, ***p < 0.001, and ****p < 0.0001.

At the distal femoral metaphysis, hypobaric hypoxia significantly increased osteoid-covered (+49%, *p* = 0.016) and osteoblast-covered surfaces (+69%, *p* = 0.021) compared with normobaric mice, while mineralizing surface, mineral apposition rate, and bone formation rate were not altered (**Figure 5**). Treatment with acetazolamide did not affect any of the dynamic bone histomorphometric parameters, the amount of osteoid-, osteoblast-, or osteoclast-covered surfaces compared with both normobaric and hypobaric mice.

Zoledronate significantly reduced mineralizing surface (–55%, *p* < 0.001 and –54%, *p* < 0.001), mineral apposition rate (–39%, *p* = 0.025 and –43%, *p* < 0.001), bone formation rate (–68%, *p* < 0.001 and –70%, *p* < 0.001), osteoid-covered surfaces (–74%, *p* < 0.001 and –82%, *p* < 0.001), and osteoblast-covered surfaces (–73%, *p* = 0.017 and –84%, *p* < 0.001) compared with normobaric and hypobaric mice, respectively. In addition, zoledronate significantly decreased all these parameters compared with mice treated with acetazolamide.

These findings suggest limited effects of exposure to hypobaric hypoxia and treatment with acetazolamide on histological indices of bone formation. In contrast, treatment with zoledronate inhibited bone formation and bone resorption.

#### 3.6.2 Cortical Bone

At the femoral mid-diaphyseal periosteal surface, no significant differences were found between any of the groups (**Table 5**).

**Table 5:** Mid-diaphyseal femoral cortical bone parameters determined by dynamic bone histomorphometry of mice housed at normobaric ambient pressure (Normo) or hypobaric pressure (Hypo) at 500 mbar for four weeks and treated with acetazolamide (AZ) or zoledronate (ZOL). Periosteal bone surface (Ps), endocortical bone surface (Ec), mineralizing surface/bone surface (MS/BS), mineral apposition rate (MAR), and bone formation rate (BFR/BS).

	Normo	Hypo	Hypo + AZ	Hypo + ZOL
Ps.MS/BS (%)	19.3 ± 11.3	23.7 ± 17.5	13.3 ± 8.80	17.1 ± 9.53
Ps.MAR (µm/day)	0.18 ± 0.28	0.43 ± 0.45	0.30 ± 0.44	0.36 ± 0.43
Ps.BFR/BS (µm^3^/µm^2^/day)	0.04 ± 0.06	0.16 ± 0.18	0.06 ± 0.09	0.08 ± 0.10
Ec.MS/BS (%)	26.5 ± 6.98	29.6 ± 7.20	23.4 ± 9.30	15.3^*,#,†^ ± 10.8
Ec.MAR (µm/day)	0.99 ± 0.78	1.17 ± 0.58	0.82 ± 0.65	0.45^#^ ± 0.57
Ec.BFR/BS (µm^3^/µm^2^/day)	0.28 ± 0.24	0.35 ± 0.19	0.45 ± 0.57	0.11^#^ ± 0.16

All data were analyzed using a one-way ANOVA, except for Ps.MAR and Ec.MAR where the non-parametric Kruskal–Wallis test was used. Data are presented as mean ± SD and n = 15–16/group. *p < 0.05 vs. Normo, ^#^p < 0.05 vs. Hypo, and ^†^p < 0.05 vs. Hypo + AZ.

At the femoral mid-diaphyseal endocortical surface, treatment with zoledronate significantly reduced mineralizing surface (–48%, *p* < 0.001), mineral apposition rate (–62%, *p* = 0.011), and bone formation rate (–69%, *p* = 0.06) compared with non-treated hypobaric mice (**Table 5**). The zoledronate-induced reduction in mineralizing surface (–42%, *p* = 0.004 and –35%, *p* = 0.042) was significantly lower compared with both normobaric mice and hypobaric mice treated with acetazolamide, respectively.

These findings suggest that the detrimental effects of exposure to hypobaric hypoxia on cortical thickness materialized earlier than the inter-labelling period of fluorochrome labels (one and two weeks before the study ended). Acetazolamide had no negative effects, while treatment with zoledronate substantially reduced endocortical bone formation.

## 4 Discussion

Exposure to high altitude is extremely challenging to most organ systems, and the ensuing cardiopulmonary adaption is vital for acclimatization. We have recently demonstrated that exposure to simulated high altitude impairs bone integrity suggesting acclimatization to high altitude exposure has detrimental effects on the musculoskeletal system (5). In the present study, mice were exposed to simulated high altitude (5,500 m) for four weeks and were treated with either acetazolamide or zoledronate.

The cardiopulmonary response to decreased barometric pressure was established by increased lung weight and right ventricle hypertrophy. As expected, lung weight substantially increased and right ventricle underwent hypertrophy in all mice exposed to simulated high altitude. These findings were accompanied by a pronounced increase in hematocrit in all groups exposed to hypobaric hypoxia and are in accordance with previous studies in rodents (4, 5, 35) and humans (24, 26, 36, 37).

We have previously demonstrated that mice exposed to simulated high altitude primarily have deteriorated cortical bone, while trabecular bone is affected less or even completely spared from any detrimental effects (5). The present study confirms that decreased barometric pressure have a negative impact on cortical bone – specifically reduced cortical thickness and increased cortical marrow area. The increased marrow area and maintained tissue area indicate that the hypoxia-induced bone resorption was located mainly at the endocortical surface. Although speculative, the observed increased marrow area and reduced cortical thickness may suggest that the hypoxia-induced enhanced erythropoiesis, seen as a substantial increased hematocrit, occurs at the expense of the adjacent cortical bone. This view is supported by Oikonomidou et al. who demonstrated a substantially reduced cortical thickness in a mouse model of polycythemia vera, and by others who showed that increased levels of erythropoietin (EPO) – the primary hormone responsible for red blood cell production – are associated with loss of bone (38, 39).

Surprisingly, the loss of cortical thickness manifested without decreased bone mineral density, bone strength, or endocortical indices of bone formation, as we have previously demonstrated in mice exposed to hypobaric hypoxia (5). In contrast to our previous study of mice exposed to simulated high altitude, the mice used in the present study were younger. Age differences between the mice from the two studies might explain some of the disparity in influence on bone mineral density and bone strength or maybe reflect an age-dependent resilience to hypobaric hypoxia. Studies in mountaineers have investigated whether age is an independent risk factor for developing AMS, but conflicting results have emerged. One study reported that younger individual more often developed symptoms consistent with AMS (40), whereas another study concluded that AMS increased with advanced age (41).

The relatively low impact of exposure to hypobaric hypoxia on trabecular bone is generally in accordance with findings from our previous study, although the effect is less pronounced in the present study. We found a non-significant trend towards an 18% decrease in trabecular bone volume fraction at L4, whereas we previously found a more pronounced significant reduction of 28% in older mice.

Acetazolamide is widely used to prevent and treat AMS in both children and adults (8). Initially, the lack of any bone protective effects of treatment with acetazolamide might seem surprising and discouraging. However, the present study did not find any substantial detrimental effects of treatment with acetazolamide, suggesting no bone safety concerns are warranted. From a bone safety perspective, acetazolamide might therefore be a preferred alternative to dexamethasone for prophylactic treatment of AMS (8, 42), since dexamethasone has a well-documented bone catabolic effect (43). In continuation hereof, it is also important to note that acetazolamide is primarily used by mountaineers for its ability to inhibit the renal carbonic anhydrase, which induces bicarbonate diuresis leading to metabolic acidosis that drives ventilation and increases oxygenation (44), thus ultimately accelerate acclimatization, and not for its ability to prevent the osteoclastic carbonic anhydrase and suppress bone resorption. Any protective effects of acetazolamide on the musculoskeletal system would therefore have been a pleasant side effect.

We did not find any effect of treatment with acetazolamide on osteoclast-covered surfaces, although others have reported a reduced number of osteoclasts and reduced osteoclastic activity in resorption assays (17). Also, acetazolamide is one of the more potent inhibitors of the osteoclastic carbonic anhydrase II compared to other less potent variants (45). Since the histological assessment resembles a snapshot of the bone surface at sacrifice only, it cannot be precluded that a reduction in osteoclast-covered surfaces occurred earlier in the study. However, this seems unlikely because treatment with acetazolamide was unable to induce any bone protective effects against hypobaric hypoxia.

Treatment with zoledronate counteracted the altitude-induced reduction of cortical thickness and prevented the expansion of the marrow area compared with non-treated or acetazolamide-treated mice exposed to hypobaric hypoxia. However, mid-diaphyseal bone strength was not increased by treatment with zoledronate, even though the bone mineral density and cortical thickness increased and the marrow area decreased compared with non-treated mice allocated to hypobaric hypoxia. This might reflect that endocortical preservation has less impact on the mid-diaphyseal bone strength than periosteal bone preservation (46). Moreover, the increased areal bone mineral density reflects the bone density of the whole femur and is therefore influenced by the increased trabecular bone volume fraction at the distal metaphysis and epiphysis. However, these skeletal sites do not contribute to the mid-diaphyseal bone strength. At L4, zoledronate increased the trabecular bone volume fraction compared with non-treated mice exposed to hypobaric hypoxia, but this finding was not accompanied by an increased bone strength. However, large variations in bone strength at L4 might have masked the effect and contributed to the discordance between microstructure and bone strength. As expected, zoledronate also decreased mineralizing surface, mineral apposition rate, bone formation rate, osteoid-covered surfaces, and osteoblast-covered surfaces. The profound impact of zoledronate on histological indices of bone formation is in agreement with previous studies by us (47, 48) and others (49, 50) and highlights its potent anti-resorptive effect. The present findings provide the first preclinical support for further clinical studies of zoledronate in the prevention of altitude-induced bone loss.

The study underlines the negative effects of exposure to hypobaric hypoxia on body weight and appetite. All mice exposed to simulated high altitude lost weight during the first week of the study and hereafter gained weight in parallel to normobaric mice. However, their food caloric intake was consistently lower throughout the study. High altitude-induced anorexia and weight loss have been demonstrated in several studies in rodents and mountaineers (51–54). Malnutrition and reduced daily caloric intake are associated with reduced bone mineral density and osteoporosis (55–57). The negative effects of simulated high altitude might, therefore, at least to some degree, be a result of the reduced caloric intake (54).

The study has several limitations. Acetazolamide was administered in the drinking water, and the target dose of 50 mg/kg/day was ensured by adjusting the concentration weekly. Weekly differences in water consumption throughout the study made it difficult to ensure the target dose was met throughout the study. The actual average acetazolamide dose (62 ± 28 mg/kg/day) was slightly above the target dose, and a rather large weekly dose dispersion was observed. The dose of acetazolamide was based on a study in rats using a dose of 50 mg/kg/day (intraperitoneal injected) and corresponded approximately to the human equivalent dose used to prevent AMS in adults (8, 58). Although challenging, we administered acetazolamide in the drinking water in order to more closely resemble how the drug is administered in mountaineers as prophylaxis for AMS and to reduce the time spent outside the hypobaric chambers as intraperitoneal injections would require daily disruption of the hypobaric environment. Moreover, sucrose was only added to the drinking water of acetazolamide-treated animals in order to encourage consumption and mask any unpleasant undertaste. However, their body weight was not affected by access to additional calories in the water compared with mice receiving tap water without added sucrose.

Another limitation is that hypobaric hypoxia was not maintained uninterrupted as is the case for expeditions to high altitude mountains. However, injections of fluorochromes to assess bone formation, cleaning and resupplying of chow and water, and weighing were performed once weekly to limit the time spent outside the hypobaric environment.

The present study investigated 16-week-old female mice only. Therefore, it cannot be ruled out that the effect of exposure to hypobaric hypoxia or the treatment response to acetazolamide and zoledronate are sex or age-dependent. However, we have recently comprehensively reviewed the effect of hypobaric hypoxia in rodents and real-world altitude exposure in mountaineers and found no indication that the associated detrimental skeletal effects are age or sex specific (54).

In conclusion, the presented study demonstrated exposure to simulated high altitude affects primarily cortical bone by reducing cortical thickness and by increasing the marrow area in young mice. Treatment with acetazolamide did not prevent cortical bone loss, whereas treatment with zoledronate did. These findings provide initial preclinical support for clinical studies of zoledronate as a potential pharmacological countermeasure against bone loss from exposure to high altitude.

## Data Availability Statement

The raw data supporting the conclusions of this article will be made available by the authors, without undue reservation.

## Ethics Statement

The animal study was reviewed and approved by the Danish Animal Experiment Inspectorate (2018–15–0201–01436).

## Author Contributions

Study design: MB, US, JT, and AB. Study conduct: MB and AB. Data collection, data analysis, and interpretation: MB, JT, and AB. Manuscript draft: MB. Figures and graphical design: MB. Manuscript revision: MB, US, JT, and AB. All authors contributed to the article and approved the submitted version.

## Funding

The study was kindly supported by Health Aarhus University, The A.P. Møller Foundation for the Advancement of Medical Science, The Frimodt-Heineke Foundation, Torben and Alice Frimodt’s Foundation, and Aase and Ejnar Danielsen’s Foundation. The funding bodies did not have any role in the study design; in the collection, analysis and interpretation of data; in the writing of the manuscript; or in the decision to submit the article for publication.

## Conflict of Interest

The authors declare that the research was conducted in the absence of any commercial or financial relationships that could be construed as a potential conflict of interest.

## Publisher’s Note

All claims expressed in this article are solely those of the authors and do not necessarily represent those of their affiliated organizations, or those of the publisher, the editors and the reviewers. Any product that may be evaluated in this article, or claim that may be made by its manufacturer, is not guaranteed or endorsed by the publisher.
